# ASSESSING BIDIRECTIONAL LONGITUDINAL ASSOCIATIONS BETWEEN SOCIAL RESOURCES AND COGNITIVE DECLINE IN LATE LIFE

**DOI:** 10.1093/geroni/igad104.0618

**Published:** 2023-12-21

**Authors:** Yun Zhang, Sean Clouston, Stacey Scott, Dylan Smith

**Affiliations:** Columbia University, New York City, New York, United States; Stony Brook University, Stony Brook, New York, United States; Stony Brook University, Stony Brook, New York, United States; Stony Brook University, Stony Brook, New York, United States

## Abstract

**Objectives:**

To better understand bidirectional longitudinal relationships between social networks and cognitive decline. Method: We examined nine waves of data from the National Health and Aging Trends Study (2011-2019). Our primary outcome was global cognition, consisting of a summation of scores on episodic memory, orientation, and executive function tests. Social network measures included measures of social participation, number and type of social ties, attributes of network alter, and neighborhood markers of social cohesion and disorder. We applied linear mixed models to study associations between time-invariant measures of social cohesion and disorder with cognitive decline over time. We employed dual-outcomes mixed models to examine the bidirectional linkages between time-varying measures of social network structures and cognitive decline. Result: Eligible participants (N = 7,325) were included at baseline and followed for an average of 4.86±3.22 years/waves. Having more friend-ties and a higher percentage of college graduates within a social network at baseline were associated with slower declines in global cognition and episodic memory. Additionally, higher global cognition at baseline was associated with smaller reductions in social network size. Less disorientation at baseline was associated with a slower decline in social participation. Social cohesion and environmental disorder were not associated with cognitive decline.

**Conclusion:**

Social networks may improve cognitive health but are also vulnerable to declining orientation. Results suggest that bidirectional longitudinal relationships link social network structure with cognitive decline and inform our understanding of the framework of associations between diverse aspects of social resources and cognitive decline in a longitudinal setting.

